# A visiting endowed chair to support mentoring and career coaching for early career researchers

**DOI:** 10.1017/cts.2023.665

**Published:** 2023-10-27

**Authors:** Lourdes E. Soto de Laurido, Walter R. Frontera, Estela S. Estape

**Affiliations:** 1 School of Health Professions, Medical Sciences Campus, University of Puerto Rico, San Juan, Puerto Rico; 2 School of Medicine, Medical Sciences Campus, University of Puerto Rico, San Juan, Puerto Rico; 3 Research Center, San Juan Bautista School of Medicine, Caguas, Puerto Rico

**Keywords:** Mentoring, coaching, research environment, career development, endowed chair

## Introduction

One of the major concerns of junior researchers is defining a career pathway that will help them become independent and funded investigators [1]. This concern is especially challenging in non-research-intensive institutions, where teaching and clinical service are top priorities and utilize most institutional resources. In addition, the availability of mentors and research coaches, one of the most critical needs of junior investigators, is particularly limited [2]. Mentoring and coaching bring together the inexperienced new investigator and the experienced senior researcher to enhance the former’s knowledge, self-confidence, and skills as they spend time together [3].

For junior faculty members who aspire to become independent researchers, developing skills to write grant applications is essential [4]. Securing external research funds is a strenuous journey that requires persistence, patience, and motivation. Access to mentorship and research infrastructure in a research-intensive-institution could facilitate the education of a new generation of independent researchers from non-research-intensive institutions who can become leaders in multicultural and multidisciplinary settings.

## Endowed Chairs

One of the common strategies to enhance the research capacity and infrastructure in a research-intensive institution is the use of an endowment to support the appointment of a chair or distinguished professor as a faculty member. This type of appointment is an academic distinction bestowed on distinguished faculty to recognize their success and contribution to the growth of an institution [5]. Its primary purpose is to recruit and retain faculty with outstanding scholarly productivity, including external competitive funding, publications, and demonstrated leadership.

So what can be done in minority institutions with limited endowment funds to attract faculty with outstanding scholarly achievements that can serve as mentors of promising young faculty? This manuscript presents an implementation of a novel concept, the Visiting Endowed Chair, as an alternative when a long-term salary commitment is not possible.

## Approach: A Visiting Endowed Chair

The University of Puerto Rico-Medical Sciences Campus is a non-research-intensive academic institution in San Juan, Puerto Rico with the HISPANICS-IN-RESEARCH CAPABILITY ENDOWMENT: School of Health Professions and School of Medicine Partnership (HiREC) [6]. HiREC is an institutional grant funded by the National Institute for Minority Health and Health Disparities, National Institutes of Health established in 2008- “*To expand and sustain high quality clinical and translational research training program and research infrastructure that will enhance minority health and increase health disparities research and enhance the diversity of the scientific workforce*.”

Supported by the HiREC Endowment, we established an Advanced Career Development Award to provide funding, coaching, and mentoring to promising Hispanic faculty researchers and help them become independent investigators. The purpose of the award is to support the research of the junior investigator. The award is $50,000 per year. It has been used to support junior faculty who are full-time faculty and 25% in Time and Effort for research, with expenses related to the infrastructure needed to complete their research projects. The selected junior investigator already has an active project and an established relationship with the proposed Visiting Endowed Chair (VEC). In some cases, they have published together, and the junior investigator already has knowledge of the institution and the research facilities of the VEC. All have been actively writing grants and submitting proposals to National Institutes of Health (NIH).

One of the cornerstones of this award is the appointment of a renowned national or international senior researcher as HiREC’s Visiting Endowed Chair to serve as the principal mentor for the investigator. The UPR officially recognizes this appointment as Visiting Endowed Chair designation in Health Disparities Research to support young faculty conducting research that may lead to their first major grant. Our definition of health disparities research is very broad, and includes multiple topics in translational research. This opens the door for “basic science” research that may be relevant to clinical problems.

The concept of a Visiting Endowed Chair is novel. It differs from the traditional Endowed Chair positions in that the latter are tenured, receive a salary, and their primary goal is to strengthen the institution’s agenda. HiREC Visiting Endowed Chair receives an official recognition by the Administrative Board of the institution and although travel expenses are covered, no salary support is provided. The VEC is expected to visit for a few days the institution, but not to relocate, at least once per year. The junior investigator (mentee) is expected to visit the institution of the VEC.

The selection of candidates to be appointed as Visiting Endowed Chair is the responsibility of the junior investigator and must fulfill the following criteria: (1) have NIH R01 level or equivalent funding, (2) national or international recognition as a health disparities researcher, and (3) strong record of scientific publications in peer-reviewed journals.

The HiREC Advanced Career Development Award and the Visiting Endowed Chair have one year of funding. The funds are intended to be used by the junior investigator only, but the VEC has access to the junior investigator’s infrastructure. The fund is for research infrastructure and does not allow for salary but will cover expenses such as travel, equipment, technical support, dissemination, and participation in scientific meetings. So far, we have funded six investigators since the beginning of the program in 2018. In terms of impact of the funding, one investigator was able to finish her research projects and was successful at obtaining an R01 grant from NIH. Other researchers are working in their R21, R34 and be prepared to submit an R01.

## Conclusions

Non-research-intensive academic institutions strengthen the research profile of their faculty by collaborating and partnering with major research institutions to become more competitive. Therefore, identifying innovative ways to support those individuals who have shown the commitment and persistence to develop a research agenda despite institutional challenges is imperative to ensure their success. The establishment of a new generation of researchers with the capacity to become leaders in multicultural, multidisciplinary settings requires institutional pathways, from formal education to institutional programs and opportunities, mentoring, and coaching. Therefore, bridge funding for the promising young investigators and having experienced funded research, such as the Visiting Endowed Chairs, are critical factors in creating a supportive research environment.
